# The Effect of Carbon Emission Taxes on Environmental and Economic Systems

**DOI:** 10.3390/ijerph19063706

**Published:** 2022-03-20

**Authors:** Zhengge Tu, Botao Liu, Dian Jin, Wei Wei, Jiayang Kong

**Affiliations:** 1 School of Economics and Business Administration, Central China Normal University, Wuhan 430079, China; zhenggetuccnu@hotmail.com (Z.T.); botaoliuccnu@hotmail.com (B.L.); dianjinccnu@hotmail.com (D.J.); weiweiccnu@163.com (W.W.); 2 Research Center of Low-Carbon Economy and Environmental Policies, Central China Norma University, Wuhan 430079, China; 3 School of Mathematics and Statistics, Central China Normal University, Wuhan 430079, China

**Keywords:** carbon emission tax, environmental quality, macroeconomic performance, evaluation index, dynamic stochastic general equilibrium

## Abstract

Carbon dioxide is believed widely to be the major contributor to global warming. Policymakers worldwide are turning to tax policies in an effort to abate carbon emissions. China is the largest emitter of carbon emissions on our planet. The central government, as well as the local official, has introduced a series of environmental regulations, such as environmental protection tax and emissions trading system, to reduce carbon emissions and improve environmental quality. In the near future, the carbon emission tax is also expected to be implemented by the Chinese government. In order to analyze and predict the effect of the carbon emission tax on environmental and economic systems, we developed a four department dynamic stochastic general equilibrium model, which includes households, enterprises, the government, and the environment. The dynamic parameters were obtained using maximum likelihood estimation. In the comparative static-s analysis, we found that after the introduction of carbon emission tax, the level in environmental quality was substantially improved, whereas most economic variables were significantly reduced. Moreover, we used impulse responses functions to evaluate how one shock to the carbon emission tax affects the steady static values for these endogenous variables in our model. We found that the carbon emission tax shock has an instantaneous effect on the majority of economic variables, but it does not affect the environmental quality immediately. In addition, we tested the Porter hypothesis and found no evidence suggesting the statement regarding this hypothesis. Finally, we applied Bayesian estimation to assure our findings in this study, again.

## 1. Introduction

Public concern about global warming and climate change has increased over the past decade [1,2,3]. Carbon emissions caused by human activities are widely believed to be mainly responsible for the increase in temperature on our planet [4,5]. Some reports point out that if the temperature on our planet continues to increase at the current rate, the global average sea level is expected to rise from 1 to 3 feet (1 m) by 2100 [6]. In an effort to solve these problems caused by climate change and global warming, policymakers all over the world are turning to tax-based policies of reducing carbon emissions [7,8,9,10].

China is the largest emitter of carbon dioxide on the earth [11]. The central government, as well as the local officials, has implemented a series of environmental regulations, such as the polluting charge and the new environmental protection tax, to abate carbon emissions [12,13,14,15,16]. In 2020, Chinese officials promised the world that China would arrive at peak carbon emissions by 2030 and achieve the goal of carbon neutrality by 2060 [17,18]. Thus, it is expected that a carbon emission tax will be introduced by the Chinese government in the not-too-distant future. However, it is critical for this policy to be rigorously analyzed before the implementation, in particular, its effect on environmental and economic systems.

In this study, we develop a four department dynamic stochastic general equilibrium model, including households, enterprises, the government, and the environment, to analyze and predict the effect of the carbon emission tax on environmental and economic systems. The steady static values for these systematic variables from four departments are compared before and after the introduction of the carbon emission tax. Our results suggest that the implementation of the carbon emission tax is beneficial for the improvement in the level of environmental quality. Meanwhile, we observe that in comparison with the scenario without a carbon emission tax, the steady static values for the majority of economic variables decline substantially after implementing the tax on carbon emissions.

In order to investigate whether, as well as how, the uncertainty surrounding the carbon emission tax affects the endogenous variables regarding the environment and economy, we then turn to evidence obtained from a variety of impulse responses. We also assume that the economy experiences one shock to the carbon emission tax with one standard deviation. Our results provide evidence that not only does the uncertainty over the carbon emission tax have an instantaneous effect on the two systems, that is, the environmental system and the economic system, but it also affects them persistently.

Several characteristics of our study distinguish ours from some existing literature on the carbon emission tax [19,20,21]. Most significantly, our study is the first to introduce environmental quality into the logarithmic utility function for a representative household. While previous studies are inclined to describe the association between households and the environment of the budget constraint at home, the inclusion of environmental quality in utility for households is particularly interesting. That is, by introducing the environmental quality into a utility function, we can calculate the steady static utility for one typical family. We also compare these steady static utilities from several contrasting scenarios, such as one without a carbon emission tax and another with a carbon emission tax.

Moreover, this study is significant since it collects data on Chinese macroeconomic variables. As we argued previously, China is the largest emitter of carbon dioxide in the world so far. If the carbon emission tax does improve the level of environmental quality through reducing carbon emissions, the variable regarding environmental quality should show a substantial increase in our model based on the Chinese data. On the other hand, if we cannot observe slight differences in environmental quality before and after the introduction of the carbon emission tax using the data from China, it is not a good choice to implement the carbon emission tax for these countries that have a similar amount of carbon emissions. In other words, in these countries, the carbon emission tax might have no effect on the level of environmental quality.

Finally, this study is different from earlier research since it includes the dynamics and randomness in our analysis [22,23]. Previous studies focused on the computable general equilibrium model, which is a static analysis that might lead to bias in the simulation of the carbon emission tax. Our study adopts the dynamic stochastic general equilibrium model, overcoming the weakness of the computable general equilibrium.

## 2. Methods

Models. In order to assess the effect of the carbon emission tax on environmental quality and macroeconomic performance, we employed an econometric model in macroeconomics; that is, the dynamic stochastic general equilibrium model. Next, we describe the model and then present our estimates.

Households. Suppose that there exist homogeneous households, whose lives are infinite, in a closed economy. The households are owners of the enterprises. They supply the labor for and gain profit from the enterprises. Moreover, the households maximize their inter-temporal utilities through consuming goods, enjoying leisure, and perceiving the environment. In classical economic theory, consumption and leisure are fundamental contributors to utility. We introduce a new variable, which describes the environmental quality that is affected by the carbon emission, into the utility function of the households.

In particular, the utility of the typical households could be described using the following function:

(1) $$U_{t}(C_{t},L_{t},Q_{t})=\bar{U}+\gamma \ln C_{t}+(1-\gamma )\ln(1-L_{t})+\varphi \ln Q_{t}$$

where $U_{t}(C_{t},L_{t},Q_{t})$ denotes the utility for one typical family at time t. $C_{t}$ is the household expenses at time t. $L_{t}$ is the labor supply at time t. $Q_{t}$ is the environmental quality. $\bar{U}$ represents the baseline utility for leisure. Here, we set it to be one unit. Additionally, γ denotes the weight of consumption in utility function, 1−γ denotes the weight of labor in utility function, and φ denotes the weight of environmental quality in utility function.

The typical households would choose the optimal amount of consumption and labor to maximize their utilities. Thus, the utility maximization problem for the typical households is defined as follows:

(2) $$\underset{\left(C_{t},L_{t}\right)}{\max}E_{t}\sum_{t=0}^{\infty }\beta^{t}U_{t}(C_{t},L_{t},Q_{t})$$

where $E_{t}(\cdots )$ denotes the mathematical expectation operator for the future variables at time t, which is subject to all the information available at time t. β represents the inter-temporal discount factor.

The budget constraint is:

(3) $$C_{t}+S_{t}\leq W_{t}L_{t}+R_{t}K_{t}$$

where $S_{t}$ denotes the saving for one representative household at time t. $W_{t}$ represents the return to labor at time t, that is, the wage at time t. $K_{t}$ is the physical capital at time t, which is used in the process of production, and $R_{t}$ is the return to capital at time t, that is, the price of capital at time t.

The capital accumulation equation is:

(4) $$K_{t+1}=(1-\delta )K_{t}+I_{t}$$

where $I_{t}$ represents the investment at time t. δ is the depreciation rate for the physical capital, which is larger than zero.

Of course, we need to set establish that the saving could be transformed into the an investment at no cost. That is:

(5) $$S_{t}=I_{t}$$

Combining Equations (1)–(5), applying the dynamic Lagrange algorithm, and allowing the first-order conditions to be equal to zero, we could obtain the expressions for consumption evolution and labor supply, which are shown as follows:

(6) $$C_{t}=\beta (R_{t}+1-\delta )C_{t-1}$$

(7) $$E_{t}=\frac{1-\gamma }{\gamma }\cdot \frac{C_{t}}{1-L_{t}}$$

Enterprises. In our closed economy, the enterprises represent the producers, which transform production factors, such as labor and capital, into final products. Following the previous assumption, we consider that the households own these factors and that the enterprises need to rent labor and capital from the households.

During the process of production, the enterprises would emit carbon dioxide into the atmosphere, which lowers the environmental quality; consequently, reducing the utility of the households. Thus, in order to prevent the environment and to improve the happiness of the households, the government would implement a carbon emission tax on the enterprises.

In addition, we assume that the carbon emission would be a proportion of the products and that the technological constraint for the enterprises obeys the Cobb–Douglas production function with a constant return to scale. That is:

(8) $$Y_{t}=AK_{t}^{\alpha }L_{t}^{1-\alpha }$$

where $Y_{t}$ denotes the products, which is also the total output in economy. A represents the productivity that is often called the total factor productivity in the related literature. α is the output elasticity regarding capital, and 1−α is the output elasticity regarding labor.

Thus, the profit maximization problem for the enterprises could be defined as below:

(9) $$\underset{\left(L_{t},K_{t}\right)}{\max}E_{t}{\sum_{t=0}^{\infty }\left(\frac{1}{1+R_{t}}\right)}^{t}\cdot (Y_{t}-W_{t}L_{t}-R_{t}K_{t}-\tau E_{t})$$

where $E_{t}$ denotes carbon emissions formed in the process of production. τ is the carbon emission tax levied on enterprises by the government.

Combining Equations (8) and (9), applying the Lagrange algorithm, and allowing the first conditions to be equal to zero, we would obtain the expressions of price for the labor and the capital, as well as the relationship between the proportional coefficient of carbon emissions and the tax on carbon emissions, which are shown as follows:

(10) $$W_{t}=(1-\eta )\cdot (1-\tau \eta )\cdot \frac{Y_{t}}{L_{t}}$$

(11) $$R_{t}=\eta (1-\tau \eta )\cdot \frac{Y_{t}}{K_{t}}$$

(12) $$E_{t}=\eta Y_{t}$$

where η represents the carbon emission coefficient, that is, the amount of carbon emissions per unit of products.

The government. In our study, the role of the government is set to reduce the carbon emission produced by the enterprises and to improve the happiness of the households. For this purpose, the government implements a carbon emission tax on the enterprises and uses the gain from the carbon emission tax to clean carbon dioxide in the atmosphere. In addition, we assume that it is balanced for the government budget over each period.

According to these settings above, the government equation could be given as follows:

(13) $$G_{t}=\tau E_{t}$$

where $G_{t}$ denotes the government revenue from tax on carbon emissions.

The environmental system. We assume that the environmental quality in the following period is dependent on the environmental quality in the current period, the carbon emission in the following period, and the government investment in clean carbon emissions, which comes from the carbon emission tax on the enterprises.

Therefore, the evolution of environmental quality could be shown using the following equation:

(14) $$Q_{t}=\bar{Q}+\theta Q_{t-1}-E_{t}+\upsilon G_{t}$$

where θ is the sustainable rate corresponding environmental quality and υ represents the ability of cleaning carbon emission for the government through tax revenue.

The resource constraint. Finally, we constrain the total budget in the closed economy as below:

(15) $$Y_{t}=C_{t}+I_{t}+G_{t}$$

Model equilibrium and stochastic shock to the economy. We have described the behavior for each department in the above. Combining these dynamic expressions, we are able to calculate the steady static values for these variables in regard to environmental and economic systems. Besides, we want to understand the effect of stochastic shocks on these systematic variables. For this purpose, we introduce additional two equations regarding the shock to tax on carbon emission and the shock to productivity, respectively, into our dynamic general equilibrium model previously explained. That is, we set τ to be $\tau_{t}$, and set A to be $A_{t}$. Meanwhile, we assume that both the carbon emission tax shock and the productivity shock follow a first-order auto-regressive process, like the ones below:

(16) $$\ln\tau_{t}=\rho_{\tau }\ln\tau_{t-1}+\varepsilon_{t}^{\tau }$$

(17) $$\ln A_{t}=\rho_{A}\ln A_{t-1}+\varepsilon_{t}^{A}$$

Data. Once our dynamic stochastic general equilibrium model has been established, in order to simulate the behaviors of environmental and economic systems, we need to assign numerical values to these model structural parameters.

We divide these parameters into two groups: one representing the static parameters and the other one representing the dynamic parameters. The former contains all the parameters from our dynamic general equilibrium model, that is {γ,φ,β,δ,α,η,θ,υ}. The latter contains these parameters of the shock to tax on carbon emissions and the shock to productivity, that is $\left\{\rho_{\tau },\rho_{A},\sigma_{\tau },\sigma_{A}\right\}$.

We calibrate the static parameters based on the previous studies. The calibrating results are shown in Table 1. According to Lu et al. [24], we set γ to be 0.50. Thus, the weight of leisure in utility function is 0.50. Following the research by Gertler and Gilchrist [25], the parameter of β is calibrated as 0.97. For the depreciation rate of capital, δ, we set it to be 0.10, which is a relatively small value [26]. Moreover, we set α, representing the output elasticity to capital, to be 0.30, and the output elasticity to labor to be 0.70, based on the study from Fischer and Springborn [27]. Besides, we separately assign 0.75, 0.99, and 5.00 to η, θ, and υ, according to the relative literature [28,29,30].

On the other hand, these estimates in dynamic parameters are obtained from maximum likelihood estimation, which is reported in Table 2. These data on the total output and the consumption between 1978 and 2018, collected from the National Bureau of Statistics of China, were used in the estimation of dynamic parameters. In order to test the sensitivity to results, we also estimate these dynamic parameters using Bayesian estimation in the Discussion. From Table 3, we can see that the auto-regressive coefficients for the carbon emission tax and the productivity are 0.89 and 0.17, respectively. This implies, after experiencing one shock to tax on carbon emissions, the environmental and economic systems would slowly return to their steady statics, compared with receiving one shock to productivity.

## 3. Results

**Comparative statics analysis.** In order to assess the effect of the carbon emission tax on environmental and economic systems, we consider two contrasting scenarios: one without a carbon emission tax, and another with a carbon emission tax. Table 3 provides the steady-state values for our system variables, respectively, for the scenarios without and with carbon emission taxes.

The first two columns of this table report the steady-state values for our model without a carbon emission tax. The steady-state value of output is 0.68. This value would be determined due to our assumption that the total time available is normalized to one unit, and that the steady-state value of productivity is equal to one unit. In addition, we see that the steady-state value of labor is 0.48. That is, 48 percent of the total time available is devoted to work. In addition, we assume that the output is equal to the sum of consumption and investment. We find that when the economic system approaches the steady state, 76 percent of income is consumed while 24 percent of income is saved. Finally, we see that the carbon emission, induced by production, is 0.51, while the environmental quality is 0.49.

The middle two columns present the steady-state values in environmental and economic variables when the carbon emission tax is introduced into our model. Here, we assume that the output of the total economy is equal to the sum of the consumption, investment, and government expenditure. We find that in equilibrium, the consumption and the investment account for the total output separately with 19 percent and 5 percent, whereas 76 percent of the total output is devoted to the government revenue. Besides, we see that the environmental quality is now 2.21, while the carbon emission is 0.28.

In the last two columns, we estimated the absolute and relative changes in endogenous variables in the scenario with a carbon emission tax relative to the scenario without a carbon emission tax by comparing Column (3) with Column (1). We find that the carbon emission tax did reduce the carbon emission and improve the environmental quality, although it might have a negative effect on the economic system: compared with the scenario without a carbon emission tax, the carbon emission level declined by 45 percentage points, which leads to an increase in the environmental quality by 1.63 unit, while the total output fell by 46 percent. According to our analysis, the carbon emission tax, introduced by the government, increases the cost of production for enterprises. To alleviate the pressure of the carbon emission tax, enterprises would lower the investment, by 88 percent, and reduce the wage, by 86 percent. Households also cut their consumption, by 87 percent, due to the reduced wage.

In sum, the carbon emission tax has a positive effect on the environmental system but a negative effect on the economic system. In particular, it could reduce the economic growth rate.

Tax change. In this section, we investigate whether the carbon emission tax rate would affect the steady-state values for our systematic variables in the scenario with a carbon emission tax. We estimated these steady-state values for a special value in carbon emission tax ranging between 0 and 1. Of course, it is possible for a particular rate of tax on carbon emissions, which is larger than 1. A tax rate larger than 1, however, would quickly decrease the total output to 0. Thus, in our analysis, the carbon emission tax is set to be equal to or less than 100 percent.

**Figure 1:**
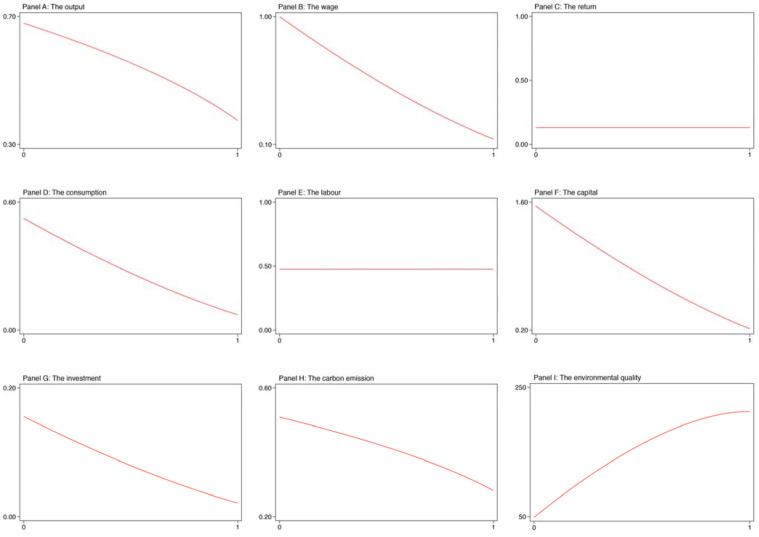
Tax change.

Figure 1 shows the results. This figure was divided into 9 panels. In panels A to C, we plot the results for the output, wage, and return, respectively. In panels D to F, we plot the results for the consumption, labor, and capital, respectively. In panels G to I, we plot the results for the investment, carbon emission, and environmental quality, respectively.

From this figure, we clearly see that the output would decrease gradually with the carbon emission tax rate rising from 0 to 1. The wage, consumption, capital, and investment also have the same trend as the output. This might be because the higher tax rate places more pressure on enterprises, which makes enterprises further reduce the desired investment and physical capital accumulation, as well as the wage that affects household income, and consequently, the expenses at home would decline with the tax rate rising. In addition, we observe that an increase in the rate of tax on carbon emission would lead to a decrease in carbon emission as well as an increase in environmental quality. This is because an increase in the carbon emission tax rate further reduces the profit of enterprises, which could slow down the product of enterprises, and consequently, the carbon emission would fall and the environmental quality would rise. Still, we find that the labor and return to capital are not dependent on the carbon emission tax rate. That is, the labor and return to capital remain constant no matter how the tax rate changes between 0 and 1.

To sum up, the rate of tax on carbon emission has a positive impact on the environmental systematic variables, but a negative impact on the economic systematic variables.

**Figure 2:**
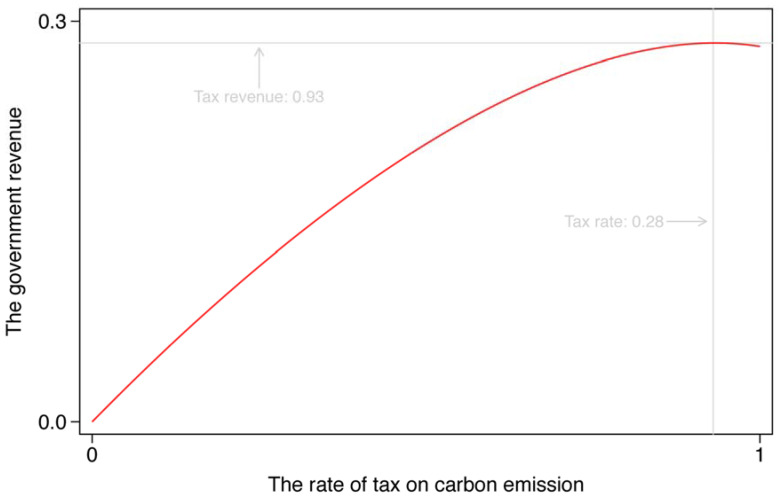
The Laffer curve.

The Laffer Curve. An important and interesting tool in tax is the Laffer curve, which describes the relationship between the tax rate and tax receipt in an economy. Here, we explore how the rate of tax on carbon emission affects government revenue, that is, fiscal revenue, based on our model with a carbon emission tax, which is shown in Figure 2.

As mentioned previously, the level of tax on carbon emission might be larger than 1. A carbon emission tax of 100 percent, however, is high enough for our analysis. From Figure 2, we can see a relatively flat curve at the starting part, where government revenue is rising with the increase in the tax rate, but with a downward trend in the end part, representing that government revenue would decline when the tax level exceeds a threshold, by 93 percent. The shape of this curve is determined by the carbon emission tax on the process of production, and hence, on the level of economic activities. Besides, we calculate the optimal tax rate regarding the maximum tax receipt for the government, which is 0.28 with a carbon emission tax of 93 percentage points.

Taken together, the relationship between the tax rate and fiscal revenue shows a typical description of the Laffer curve. We do not recommend the government to set a relatively high tax rate of carbon emission, however, even if the tax rate does not exceed the threshold, over which the government revenue would decline rapidly. This is because compared with a low level of tax, the product of enterprises and consumption of households at a high tax level are lower, implying that the total social welfare would decrease when the government increases the tax rate.

Carbon tax uncertainty. So far, we have assessed the effect of the carbon emission tax on environmental and economic systems based on several contrasting scenarios. However, one aspect, which needs particular attention, is that these analyses above are based on a determined model. That is, we do not take into consideration the uncertainty surrounding the carbon emission tax in the aforementioned results. In order to fill the gap, thus, we introduce the exogenous shock to the carbon emission tax into our dynamic general equilibrium model, known as the dynamic stochastic general equilibrium model in the related literature, and analyze the effect of the shock to tax on endogenous environmental and economic variables.

**Figure 3:**
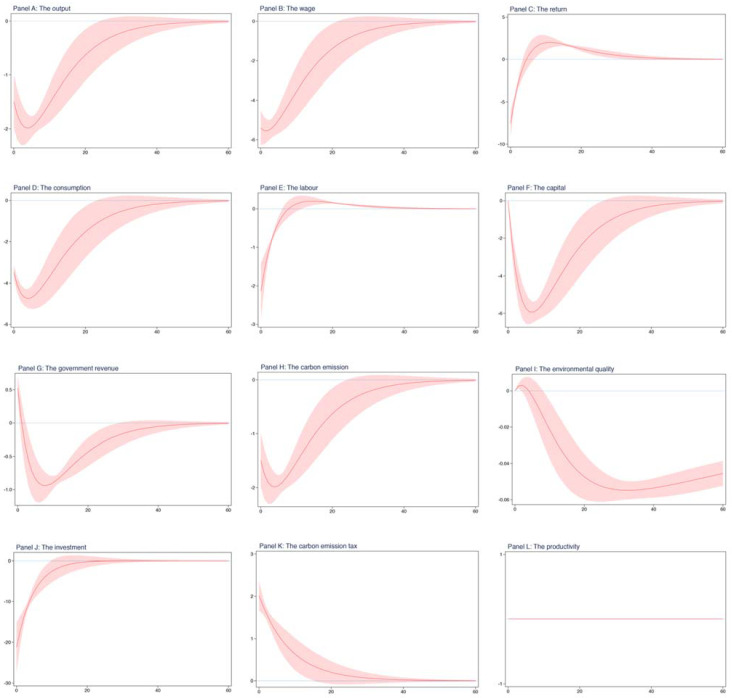
The impulse responses from maximum likelihood estimation.

These results are shown in Figure 3, which is structured into 12 panels. The top 3 panels separately plot the results for output, wage, and return. The middle section contains 6 panels, which plot the results for consumption, labor, capital, government revenue, carbon emission, and environmental quality, respectively. The bottom 3 panels separately plot the results for investment, carbon emission tax, and productivity. In each panel, the horizontal axis represents time, which plots the 60 periods after the shock to the carbon emission tax, and the vertical axis represents the change in one unit relative to the steady-state values regarding the variables. The red line depicts the dynamics of endogenous variables, and the red shadow depicts its 95 percent conference interval.

From Figure 3, we can see that not only did the shock to the carbon emission tax have an instantaneous effect on the majority of systematic variables but it also did persistently. Given an increase in the carbon emission tax with one standard deviation in equilibrium, the persistence is due to the process of production.

First, we see that the output decreases immediately, falling below its steady-state value, and continues to decline. After about 5 periods, the negative deviation begins to shrink but lasts for a relatively long time, and approximately, in the 40th period, the output returns to the steady-state value. The fact observed here is caused by two factors. One is the persistence of the carbon emission tax shock and the other is the persistence of the production process. Therefore, as we expect, one positive shock to the carbon emission tax has a negative effect on the output.

Second, in comparison with its steady-state value, the consumption also falls instantaneously, but declines to a larger extent than the output. Afterwards, the negative deviation continues to increase, while after about 5 periods, the consumption begins to rise, and approximately, in the 40th period, the consumption returns to its steady-state value. Comparing panel D with panel A, we find that the trends in the consumption and the output are very similar. This is because the expenses at home come from the sum of the return to labor and return to capital, which are determined by the decision on enterprise production, and consequently, the consumption and the output exhibit an analogous evolution. As we expect, there is a dramatic decline in the labor and the investment as a result of the shock to the carbon emission tax, but afterwards, the investment quickly increases to its steady-state value, while the labor first rises above its steady-state value and then falls to its steady-state value. However, the carbon emission tax shock does not seem to have an immediate effect on the physical capital but has a persistent effect. For example, during the period between 0 to 7, the capital is on the slide, while afterwards, the capital begins to increase, and approximately, in the 50th period, the capital returns to its steady-state value. Of course, we observe that the government revenue exhibits an instantaneous growth since one positive shock to the carbon emission tax improves the tax receipt, which is the only source of government revenue.

Third, for the price of production factors, both the wage and return to capital have an immediate decline, with a relatively large proportion, resulting from the reduction in enterprises production with regard to one standard deviation positive shock to the carbon emission tax. On the other hand, both of them have different dynamic paths. For example, after experiencing the shock to the carbon emission tax, the wage earlier decreases steadily and later increases rapidly, while the return to capital earlier quickly rises above its steady-state value, and later gradually falls to its steady-state value, as a result of the persistence of the shock to the carbon emission tax.

Finally, regarding our environmental variables, that is, carbon emission and environmental quality, we find that the shock to the carbon emission tax has an instantaneous effect on the carbon emission but has no immediate effect on the environmental quality. Besides, the persistent effects on both of them are distinguished. For example, during the starting period, the carbon emission would continue to decrease, arriving at the lowest point, and then bounce back to its steady-state value. The environmental quality would increase a little in the starting period, decline substantially in the middle period, and rise slowly, reaching a new steady-state value.

Overall, our results show that one shock to the carbon emission tax would not only affect the majority of our systematic variables instantaneously but also have a persistent effect on them. In particular, the shock to the carbon emission tax immediately reduces the enterprises’ products and expenses at home and improves the environmental quality level.

## 4. Discussion

**Robustness to estimation approach.** Previously, these impulse response results were obtained based on the estimated dynamic parameters, which are from maximum likelihood estimation. Following the related literature, these parameters in regard to the exogeneous shock could also be obtained using the Bayesian estimation approach. In this section, we apply the Bayesian rule to estimate these dynamic parameters regarding the carbon emission tax and productivity and check whether the impulse response results are not sensitive to the estimation approach.

**Figure 4:**
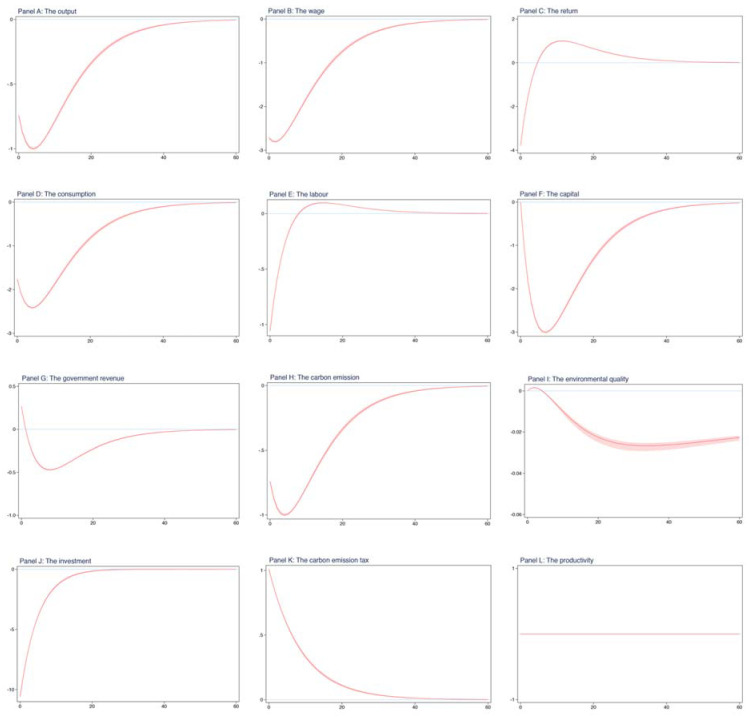
The impulse responses from Bayesian estimation.

Table 4 presents the results for these dynamic parameters. In comparison with Table 2, we can see that the two autoregressive coefficients for the carbon emission tax and productivity obtained from Bayesian estimation are very close to those using maximum likelihood estimation. However, the estimates in standard deviations of exogenous shocks, from the Bayesian and the Frequentist, are significantly distinguished. For example, in Table 4, the estimated standard deviations of the carbon emission tax shock and productivity shock are about 1, while those in Table 2 are about 2. In other words, for the same shocks to the carbon emission tax and productivity, the estimates obtained from maximum likelihood estimation are likely two times larger than the estimates from Bayesian estimation, suggesting that there could be differences between both results regarding two different estimation approaches. We also depict the dynamic paths for environmental and economic systematic variables as a result of one shock to the carbon emission tax, which is shown in Figure 4.

Regarding Figure 3, Figure 4 is divided into nine panels. The top section contains three panels, which plot the impulse responses for the output, wage, and return to capital, respectively. The middle section contains six panels, plotting the impulse responses for the consumption, labor, physical capital, fiscal revenue, carbon emission, and environmental quality, respectively. The bottom section contains three panels that plot the impulse responses for the investment, carbon emission tax, and productivity, respectively.

From Figure 4, we see that one shock to the carbon emission tax, with one standard deviation, instantaneously decreases all the economic endogenous variables except for the fiscal revenue and physical capital, such as the output and consumption. Meanwhile, the carbon emission tax shock has an instantaneous effect on carbon emission, which decreases the level of carbon emission.

These results in Figure 4 are very similar to those in Figure 3. That is, not only does one shock to the carbon emission tax immediately affect the majority of systematic endogenous variables but it also has a persistent effect on these variables. This gives us confidence that our results are robust to the alternative estimation approach.

Welfare analysis. Referring to our previous assumption, households are the owners of production factors, such as labor and capital, which the enterprises need to rent; the profit is zero in a perfectly competitive market, and the households make a decision to maximize their utilities. Therefore, here, we consider the utility of a representative family as the tool in the analysis of welfare.

Following the analysis in Table 3, we first calculated the utility of a typical household in equilibrium, under the scenario without a carbon emission tax, which is about 1.12 units. Then, we estimated the steady-state utility in the carbon emission tax case scenario, that is, approximately, 0.41 units. We find that the total utility declines, by 63 percentage points, when the government imposes a proportion levy on carbon emissions produced during the process of production. As shown in Table 3, not surprisingly, the stationary consumption also rapidly decreases due to the implementation of a carbon emission tax, which is a contributor to the decline in the total utility. In addition, we observe that even though there is a substantial improvement in the environmental quality, the share of the environmental quality of the utility function is relatively small. The observed fact that the total utility is in equilibrium, thus, is probably because the increase in utility due to the improvement in the environmental quality is far lower than the decrease in utility due to the reduction in consumption.

The results of the welfare analysis have some important implications for policy-relevant issues. For example, the government imposes a carbon emission tax on enterprises, which would transform the tax to households, reducing home expenses, and as a consequence, the total utility falls. Therefore, it is worth considering whether the government should tax carbon emissions. Besides, if the carbon emission tax is implemented by the government, we recommend that the government pass a proportion of tax revenues to the households so that it can strongly make up for the loss in consumption, and consequently, boost the steady-state utility.

The Porter hypothesis. The Porter hypothesis means that a moderate environmental regulation could improve the environmental quality, and at the same time, be able to increase the output by encouraging innovation and promoting originality.

There are an increasing number of studies suggesting the Porter hypothesis. In our study, however, we found no evidence of the Porter hypothesis. For example, in Table 3, we see that after introducing the carbon emission tax into our model, the majority of economic variables experience a dramatic decline, even if the level of environmental quality has an improvement and the carbon emission reduces significantly. Additionally, from Figure 3 and Figure 4, we found that one shock to the carbon emission tax has no instantaneous effect on the environmental quality, while it substantially decreases the output and consumption, which is completely opposite to the statement regarding the Porter hypothesis.

Overall, our results minimally suggest the Porter hypothesis. This is in contrast with findings from the aforementioned studies. A possible explanation is that we adopted the dynamic stochastic general equilibrium model to simulate several contrasting scenarios, such as one without a carbon emission tax and the other with a carbon emission tax, whereas they collected related information from open databases to perform a regression analysis. Although we cannot say which approach is better and more suitable for an empirical study, the simulation method would have a more direct and clear causal interpretation than the regression analysis, because the latter has more confounding factors that need to be considered in practice, which is difficult to include in the regression analysis in order to reduce the confounding effect.

Of course, further to our earlier comments, the simulation results rely heavily on the structure of the model. Thus, future studies, which can develop the dynamic stochastic general equilibrium model that is different from our model, are needed to confirm our findings, as well as to test the Porter hypothesis again.

## 5. Conclusions

The planetary population is expected to arrive at 8.5 billion by 2030 and exceed 9.7 billion by 2050. The rapid and substantial growth puts more pressure on the environment, in particular, global warming which is mainly caused by carbon emissions due to human activities, such as production and business. Faced with this huge challenge, not surprisingly, more and more policymakers are turning to tax policies of abating carbon emissions such as the carbon emission tax. It is important, however, for the carbon emission tax to analyze and predict its effect on environmental and economic systems.

In this paper, we proposed a dual systems dynamic stochastic general equilibrium model which contains four departments that are households, enterprises, the government, and the environment. Using maximum likelihood estimation, we obtained the estimates in dynamic parameters. Combining these dynamic parameters and static parameters, which are calibrated according to the aforementioned studies, we investigated the change in environmental and economic systems, such as the environmental quality, the total products, and the expenses at home, after the introduction of a carbon emission tax by the government. Moreover, we explored the effect of one shock to the carbon emission tax on endogenous systematic variables, by impulse responses functions. Besides, we used the Chinese macroeconomic data to test the Porter hypothesis.

Our findings in this study are devoted to the continuing argument regarding the carbon emission tax and environmental quality, as well as economic growth. Our results show that the carbon emission tax increases the environmental quality, but also decreases the output and consumption, suggesting that the implementation of a carbon emission tax is beneficial to the improvement in environmental quality at the cost of slowing economic development. This finding is in line with an earlier study that reported a positive correlation between environmental protection tax and environmental quality, and a negative correlation between environmental protection tax and economic growth. Our results also illustrate that one shock to the carbon emission tax immediately negatively affects the majority of economic variables, as well as the carbon emission level, whereas it has no instantaneous effect on environmental quality. This finding is partially consistent with studies providing evidence on the negative effect of tax shock on economic development. Differences in the immediate effect on environmental quality, on which the aforementioned studies reported a dramatic increase in environmental quality when environmental systems receive a shock to the carbon emission tax, are probably due to the different estimates of dynamic parameters. An alternative explanation might be the distinguished structure of the dynamic stochastic general equilibrium model. For example, in their model, there exists an additional department of energy. In addition, our study provides contrasting evidence on the statement regarding the Porter hypothesis. That is, whether we introduce a carbon emission tax into our model or the economy receives a shock to the carbon emission tax, both of the results exhibit a substantial decline in output. However, according to the Porter hypothesis, the output should be increased as a result of moderate environmental regulation. Therefore, more studies are needed to confirm our findings using an analogous dynamic stochastic general equilibrium model.

Our study has limitations. Its ecological nature, which is based on the dynamic stochastic general equilibrium model and the data at an aggregate level, means that these findings are not able to describe individual behavior. For example, we observe that the expenses at home experience a remarkable decline after the introduction of the carbon emission tax from our model. In fact, however, different individuals could have a contrasting response to a tax on carbon emissions. Some people would reduce their consumption, while others might keep a constant amount of consumption due to their relatively strong consumption habits. Moreover, our study cannot establish a causal relationship for the systematic endogenous variables, although our results have a causal interpretation for the effect of the carbon emission tax on environmental and economic variables. We offer some explanations in this study, based on the existing economic theory. However, future research should try to explore the causality among these macroeconomic variables after the implementation of a carbon emission tax, in order to better understand the effect of a carbon emission tax on environmental and economic variables. In addition, our results are highly dependent on the structure of the model. In other words, once the structure of the model is changed, such as adding other departments into our model, it is very possible, for the results obtained from the new model, to be different from the results from the original model. We make a comparison of results between our model and other models, suggesting that most of the findings are consistent; however, we cannot assure that each dynamic stochastic general equilibrium model discussing the effect of carbon emission tax draws the same conclusion.

Taken together, our results suggest that the carbon emission tax has a significant positive and robust effect on the environmental system, but a substantial negative effect on the economic system. Future analysis should consider adding an energy department in our model and confirm our findings in this study as well as test the Porter hypothesis, again.

## Figures and Tables

**Table 1:** Calibrated parameters obtained from the previous literature.

Parameter	Value	Parameter Description
γ	0.50	The share of consumption in utility function
β	0.97	The inter-temporal discount factor
δ	0.10	The capital depreciation rate
α	0.30	The output elasticity in relation to capital
η	0.75	Carbon emissions per unit of Gross Domestic Product
θ	0.99	The growth rate of environmental quality
υ	5.00	Decontamination of carbon emissions through government expenditure

Note: This table presents static parameters and their corresponding sources.

**Table 2:** Parametric estimates obtained from maximum likelihood estimation.

Parameter	Parameter Description	Mean	Standard Error
$\rho_{\tau }$	The shock to tax	0.89 ***	0.03
$\rho_{A}$	The shock to productivity	0.17 ***	0.06
$\sigma_{\tau }$	Standard deviation of tax shock	2.01 ***	0.18
$\sigma_{A}$	Standard deviation of productivity shock	2.22 ***	0.10

Note: This table presents estimates of dynamic parameters, *** *p* < 0.01.

**Table 3:** Steady state values for the economic and environmental systems in different scenarios.

Sign	Description	No Policy		Carbon Emission Taxes		Absolute Change	Relative Change
		Value	Ratio to Y	Value	Ratio to Y	(3)-(1)	(5)/(1)
		(1)	(2)	(3)	(4)	(5)	(6)
Panel A: Economic system							
Y	The total out put	0.68	1.00	0.37	1.00	−0.31	−0.46
W	The wage of labor	1.00	-	0.14	-	−0.86	−0.86
R	The return to capital	0.13	-	0.13	-	0.00	0.00
C	The consumption	0.52	0.76	0.07	0.19	−0.45	−0.87
L	The labor	0.48	-	0.48	-	0.00	0.00
K	The capital	1.56	2.29	0.21	0.57	−1.35	−0.87
I	The investment	0.16	0.24	0.02	0.05	−0.14	−0.88
G	The government revenue	-	-	0.28	0.76	-	-
Panel B: Environmental system							
E	The carbon emission	0.51	-	0.28	-	−0.23	−0.45
Q	The environmental quality	0.49	-	2.12	-	1.63	3.33

Note: The table presents the steady static values. The reported values on the environmental quality, in columns (1), (2) and (5), are divided by 100, which does not affect the calculation of relative change in this variable, that is, the value in column (6). Panel A reports the results of economic system. Panel B reports the results of environmental system.

**Table 4:** Parametric estimates obtained from Bayesian estimation.

Parameter	Parameter Description	Mean	Standard Deviation
$\rho_{\tau }$	The shock to tax	0.90	0.002
$\rho_{A}$	The shock to productivity	0.17	0.002
$\sigma_{\tau }$	Standard deviation of tax shock	1.01	0.006
$\sigma_{A}$	Standard deviation of productivity shock	1.01	0.004

Note: This table presents estimates of dynamic parameters.

## Data Availability

Correspondence and requests for materials should be addressed to Jiayang Kong.
